# Fusion of NSGA-II and Latin hypercube sampling for optimizing node displacement in thin-film IME molding

**DOI:** 10.1038/s41598-025-33062-y

**Published:** 2026-02-14

**Authors:** Hanjui Chang, Fei Long, Jiaquan Li

**Affiliations:** 1 Department of Mechanical Engineering, College of Engineering, Shantou University, Shantou, 515063 China; 2 Intelligent Manufacturing Key Laboratory of Ministry of Education, Shantou University, Shantou, 515063 China

**Keywords:** Node detection NSGA-II model, Latin hypercube sampling (LHS), Thin-film IME technology, Firefighter magnetic levitation shield, Circuit damage, Process parameters

## Abstract

In the high-risk environment of firefighting operations, the safety of firefighters depends on the performance of advanced protective equipment. This paper proposes a magnetic levitation shield that combines thin-film in-mold electronic (IME) forming technology and magnetic levitation control as protective equipment. Its non-contact and high strength characteristics ensure the safety of firefighters. However, the execution of the shield’s protective function is inseparable from the precise control of the magnetic levitation system, so the defects in the circuits formed by the IME need to be controlled to minimize the loss of current and power as much as possible, thereby improving the stability of the shield. Since the degree of defect in the IME circuit has a greater correlation with the node displacement of the product itself, the main objective of this study is to minimize the node displacement of the product by setting different injection molding parameters. Therefore, this paper innovatively combines the non-dominated sorting genetic algorithm II (NSGA-II) with Latin Hypercube sampling (LHS), and combines Moldex 3D to optimize and obtain different Palatto solutions, and finally selects the eligible combination of process parameters based on the research purpose. Through research and discussion, it was found that the synergistic effect of the two led to an optimization rate of 65.7–89.7% for node displacement. Finally, through the analysis of the impact of different degrees of circuit damage caused by different degrees of node displacement on current loss and power loss, the effectiveness of the method was demonstrated in multiple aspects, providing effective safety guarantee for workers in high-risk environments. The research results not only provide a technical solution and theoretical basis for the design, manufacture and maintenance of magnetic levitation shields for firefighters, but also promote the cross-application of in-mold electronic technology and intelligent optimization algorithms, opening up a new path for improving the safety protection level of firefighters in high-risk environments.

**Supplementary Information:**

The online version contains supplementary material available at 10.1038/s41598-025-33062-y.

## Introduction

Firefighters often face life-threatening risks while on duty. Among these dangers, falling objects pose a significant threat to their safety. Significant progress has been made in magnetic levitation, in-mold electron (IME) film technology and polymer materials in recent years. Taking advantage of these technological advancements, this paper presents an innovative concept: to create wearable armor with built-in circuit functions for firefighters by integrating in-mold electronic film technology, injection molding process and magnetic levitation system based on polymer materials. Meanwhile, by injection molding the polymer material, a high-strength, lightweight shield with embedded magnets was created. When the armor and shield work together, an innovative magnetic levitation protection system is formed. Because of the interactivity of the in-mold electronic film, firefighters can control the current of the entire system through keypress operations, allowing the shield to adjust its position according to the current changes. The non-contact nature of the system provides firefighters with greater protection in a variety of dangerous scenarios.

Previous research has laid an important foundation for the field. In 2020, Lee^1^ developed a highly stretchable conductive ink for 3D in-mold electronics. By combining screen printing with injection molding, this method integrates hard coating lamination and electronic device packaging. During the high temperature and high pressure molding process, conductive fillers are compacted and encapsulated, significantly enhancing conductivity. The results show a high degree of process compatibility, providing an efficient manufacturing solution for wearable devices. In 2010, Takao^2^ addressed the issue of insufficient levitation by adding ferromagnetic plates and iron rods to the superconductor, thereby increasing the levitation height. However, existing in-mold electronics research has mainly focused on enhancing functionality by developing new ink materials and combining them with injection molding. Research on magnetic levitation often relies on adding external elements, such as ferromagnetic plates, to increase the levitation height, but these methods lack flexibility and are difficult to adjust. In contrast, this study takes advantage of the unique properties of the in-mold electronic thin film to adjust the device current through user interaction, allowing the shield to move flexibly within the maglev system.

However, to take full advantage of the electrical properties of the in-mold electronic film requires a high degree of consistency in its internal circuit routing. This is closely related to defect problems in the molding process of plastic parts, such as node displacement and volume shrinkage. In this study, we explicitly prioritize minimizing node displacement over volume shrinkage. This decision is based on the functional requirements of the application: node displacement directly causes physical stretching, compression, or distortion of the IME circuits attached to the product surface. This circuit deformation irreversibly alters the conductor’s cross-sectional area and path length, and can even lead to insulation layer damage and short circuits. According to Ohm’s law, this directly results in unstable current transmission and a sharp increase in power loss, ultimately causing instability in the maglev system, which relies on a stable current, posing a direct threat to firefighter safety. In contrast, uniform volume shrinkage generally has a secondary impact on circuit integrity. Therefore, controlling node displacement is the most critical factor for ensuring the functional reliability of the entire maglev protection system.When the nodes of the armor shift or shrink in volume, the attached in-mold electronic film circuits also move along, causing the current in the device to be unstable. As a result, the entire suspension system becomes unstable and fails to provide effective protection for the firefighter. Since the defects of injection-molded products largely depend on the injection process parameters, and different combinations of parameters have a very different impact on the armor, there is an urgent need for a method to minimize node displacement and volume shrinkage.

Latin hypercube sampling (LHS) has been proven to be an efficient alternative to mesh search when adjusting the parameters of deep learning models, as it can generate training data and build response surface models. The non-dominated sorting genetic algorithm (NSGA-II) excels at balancing multiple objective functions to obtain the Pareto solution set, providing researchers with flexibility in choosing the optimal solution.

**Fig. 1:**
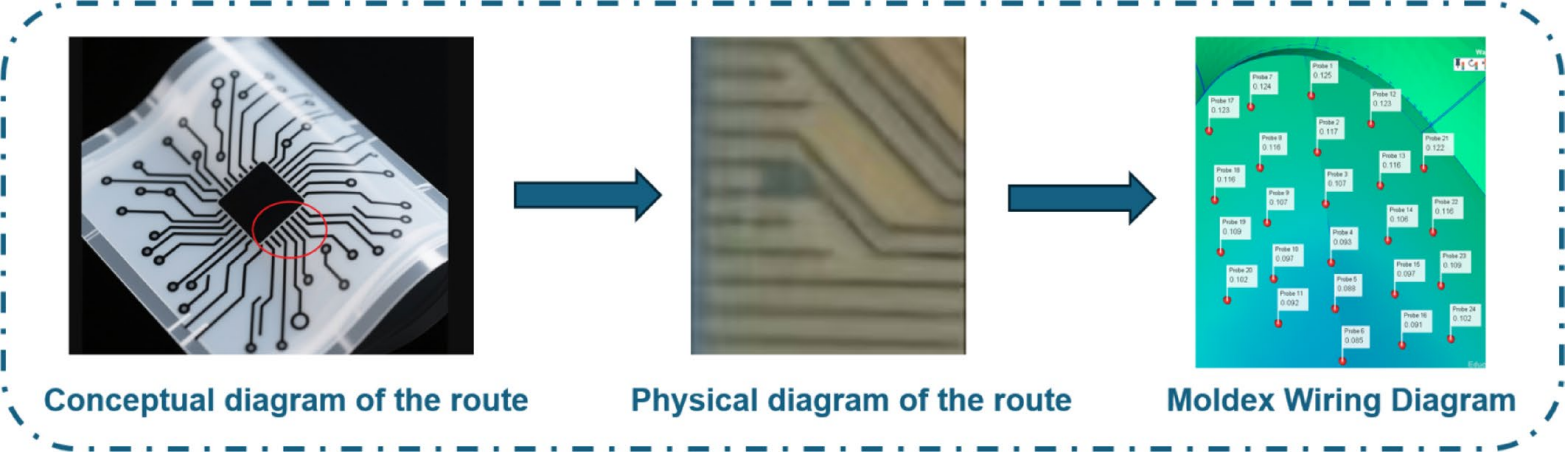
Conceptual roadmap for node displacement analysis.

In this study, Latin Hypercube sampling (LHS) and non-dominated sorting genetic algorithm (NSGA-II) were combined to study and optimize parameter combinations in order to solve the problem of minimizing node displacement and volume shrinkage of armor, with the aim of obtaining the Pato solution set. This approach ensures the minimization of armor defects, stabilizes circuit functions, and ultimately provides protection for firefighters. The case study demonstrated that the combination of Latin hypercube sampling and the non-dominated sorting genetic algorithm effectively reduced the values of the two target quantities, verifying the functionality and practicality of the proposed method (As shown in Figure 1).

## Literature review

### Effects of injection molding parameters on IMD film properties

In-mold decoration (IMD) technology, which integrates printed films with injection molding processes, has become a pivotal surface treatment method in consumer electronics, automotive, and appliance industries. The quality of IMD products is governed by complex interactions among multiple process parameters, with researchers employing both experimental and simulation approaches to unravel these relationships.

In terms of film mechanical properties, parameter optimization requires balancing competing objectives. Meng’s^3^ investigation using Taguchi orthogonal experiments revealed that increasing melt temperature (240-280°C) enhanced pattern layer wear resistance by 40% but reduced bonding strength by 15%, while holding pressure (60-100 MPa) contributed up to 65% to bonding strength . This trade-off between wear resistance and adhesion highlights the need for synergistic parameter control, which Meng addressed by optimizing melt temperature (265°C) and holding pressure (80 MPa) to reduce pattern shedding rates.

Thermal management at the film-mold interface presents another critical challenge. C. H. Wu’s^4^ comparative study of PC and PET films demonstrated that PC films reduced mold surface temperature by up to 17.7°C, 32% higher than PET films, while each 0.1 W/(m·K) increase in thermal conductivity caused 22.9°C fluctuations at the interface. By implementing film preheating (95°C) combined with high thermal conductivity mold steel, Wu successfully controlled interface temperature fluctuations within ±5°C, effectively mitigating film shrinkage and cracking issues.

Product deformation control requires addressing asymmetric cooling effects. Lee’s^5^ research on U-shaped components found that 90° corner pieces warped 1.2mm, three times that of 150° pieces, with corner angle (48% contribution) and melt injection pressure (32% contribution) identified as main influencing factors. The insulating effect of decorative films reduced cavity-side cooling rates by 60%, leading to asymmetric shrinkage. Through large fillet designs (150°) and maintaining mold temperature differences within 5°C, warpage was reduced by 23%.

For printed layer durability, Liu^6^ established a process window model based on shear stress thresholds for glass fiber-reinforced PET systems. The study found that 1mm thick specimens exhibited 40% larger ink shedding areas than 2mm specimens, with melt temperatures above 280°C increasing scouring areas by 65% as shear stress exceeded the critical 0.8 MPa. By limiting melt temperature to 265°C, raising mold temperature to 85°C, and implementing low-speed injection (50 mm/s), shear stress was reduced by 42% and ink retention rates increased to 92%.

Surface quality optimization for complex geometries has been addressed through innovative forming strategies. Chen’s^7^ research on polycarbonate films demonstrated that forming at 160°C produced surface roughness Ra of 3.2μm, four times that at 147°C . Through stepwise molding (147°C preforming + 160°C final forming) with 0.3 MPa vacuum pressure, fiber penetration rates in 5mm radius curvature regions were reduced from 35% to 8%, increasing qualified rates for complex parts from 45% to 82%. Further thermoforming studies revealed that each 10°C increase in mold temperature increased sidewall thinning rates by 12%, while multi-step pressure application processes improved thickness uniformity by 28%^8^.

Deep-cavity structure formation benefits from staged processing approaches. Puentes^9^ found that 0.125mm PC films achieved 220% sidewall stretching rates at 110°C mold temperature, 18% higher than at 90°C, but preheating above Tg+30°C (150°C) increased thickness variation coefficients by 25%. Implementing three-stage forming processes (30-50-70 kPa pressure, 50% extended total time) reduced wrinkling rates for 40mm deep cup pieces from 35% to 12% and improved thickness uniformity by 40% at 120 mm/s punch speeds.

Collectively, these studies demonstrate that IMD process quality depends on nonlinear couplings among thermal, pressure, and temporal parameters. While traditional IMD technology excels in decorative applications, its limitations in functional integration have prompted the development of in-mold electronics (IME) technology, which embeds sensors and circuits directly into film layers, transforming in-mold forming from static decoration to dynamic interaction.

### Applications and advantages of NSGA-II and LHS in multi-objective optimization

The integration of Latin Hypercube Sampling (LHS) and Non-dominated Sorting Genetic Algorithm II (NSGA-II) has emerged as a powerful paradigm for addressing complex multi-objective optimization problems across engineering domains. This methodology combines LHS’s efficient parameter space exploration with NSGA-II’s ability to identify well-distributed Pareto-optimal solutions.

In building environmental optimization, Wu^10^ addressed the challenge of simultaneously optimizing energy consumption, daylighting, and thermal comfort in residential buildings ^10^. By employing LHS for spatially uniform sampling of building envelope parameters and constructing accurate prediction models through Bayesian-optimized XGBoost, the study effectively handled multi-objective trade-offs using NSGA-II, obtaining Pareto solution sets with superior overall performance compared to traditional single-objective approaches.

Energy equipment design has benefited from this integrated approach in addressing material-heat transfer conflicts. Wang^11^ focused on tree-shaped finned thermal energy storage units, where LHS enabled low-cost, efficient sampling in high-dimensional parameter spaces while NSGA-II achieved synchronous improvement of material utilization and heat transfer efficiency through multi-objective collaborative optimization . The constructed response surface models provided effective guidance for structural optimization, demonstrating the method’s advantage in balancing competing design objectives.

Electronic thermal management presents challenges requiring efficient design space exploration. Gupta^12^ generated parameter combinations for pore structures and flow conditions using LHS, ensuring sample representativeness in the input feature space . Computational fluid dynamics simulations provided training data for neural network surrogate models, with NSGA-II subsequently identifying optimal thermal performance solutions in complex design spaces, overcoming the efficiency limitations of traditional trial-and-error methods.

Wear control in mechanical systems has been enhanced through this methodology. Zhang^13^ addressed severe wear problems in pipeline fittings by discretizing the structural parameter space using LHS and establishing Kriging surrogate models based on discrete element simulation data . NSGA-II enabled synchronous optimization of average wear rates and maximum wear amounts, achieving multi-objective collaborative optimization that significantly extended service life while reducing experimental costs.

Complex system engineering applications demonstrate the method’s versatility. In ship engineering, Liu BL^14^ selected hull form parameter combinations through LHS, established Kriging approximation models, and applied NSGA-II for multi-objective optimization. The approach ensured spatial uniformity in parameter sampling while finding optimal balance points among drag reduction objectives, significantly improving navigation efficiency. Similarly, Yu^15^ tackled strong coupling challenges in motor systems through LHS-based stratified sampling of sensitive parameters and combined surrogate models with NSGA-II optimization, effectively handling complex trade-offs among electromagnetic performance metrics and enhancing overall motor performance.

Lightweight protective structures represent another successful application domain. Carakapurwa^16^ generated cellular structure parameters using LHS and trained neural network surrogate models with finite element simulation data. NSGA-II identified optimal balances between lightweight characteristics and energy absorption targets for battery protection systems, with LHS effectively covering the complex design space to ensure comprehensive optimization.

The consistent success of LHS-NSGA-II integration across these diverse domains stems from its fundamental advantages: LHS provides high-quality training data for surrogate models through spatially uniform sampling with minimal computational cost, while NSGA-II leverages the computational efficiency of these models to conduct thorough multi-objective optimization. This "efficient sampling—precise modeling—multi-objective optimization" paradigm has proven particularly valuable for problems involving computationally expensive simulations or physical experiments, where traditional approaches face prohibitive computational burdens.

### Research progress in magnetic levitation systems and node displacement control

Recent advancements in magnetic levitation technology and injection molding process optimization have created new opportunities for protective equipment development, with researchers addressing stability challenges through material innovations, structural optimization, and advanced control strategies.

Magnetic levitation system stability has been enhanced through multiple approaches. Takao et al.^17^ improved levitation stability by replacing permanent magnets with high-temperature superconducting (HTS) magnets and optimizing magnetic field distribution through adjusted HTS magnet-block distances. Murakami’s^18^ team addressed repulsive maglev stability limitations by introducing the rigidity characteristics of superconducting magnetic bearings and utilizing superconducting pinning effects to counteract unstable forces, achieving stable suspension without active control while maintaining high levitation buoyancy. For system stability assessment, Bernstein’s^19^ research group established coupled calculation models for lateral and levitation forces by analyzing mechanical characteristics of superconducting magnetic levitation hybrid devices, while Sahoo’s^20^ team developed multimodal control architectures optimized through evolutionary algorithms, achieving robustness breakthroughs in levitation devices.

Injection molding defect control has evolved toward intelligent optimization methods. For molding defects caused by barrel screw offset, Chang et al.^21^ proposed virtual IMD overlay technology based on polyetheretherketone (PEEK) films, enabling rapid diagnosis and parameter optimization through mold quantification and steady-state simulation. Addressing node displacement and residual stress issues in brain-computer interface integration, research teams employed multi-strategy differential evolution algorithms with Latin hypercube sampling, reducing node displacement from 0.585 mm to 0.027 mm (95.38% optimization rate) while ensuring circuit reliability^22^. For damage prediction and repair, Chan developed integrated detection-repair solutions combining IMD film rupture characteristics with mold indexing for damage localization, COMSOL and Johnson-Cook stress models for impact deformation simulation, and additive manufacturing for functional recovery^23^.

These advancements demonstrate a clear technological trajectory: magnetic levitation research has progressed through coordinated developments in materials, structures, and control strategies, while injection molding optimization has embraced parameter mapping, intelligent algorithms, and integrated detection-repair technologies. The convergence of these fields through in-mold electronics (IME) technology represents a significant innovation, embedding sensors and circuits into film layers to enable intelligent interaction through injection molding. The application of NSGA-II and LHS algorithms to injection molding parameter optimization has proven particularly effective in simultaneously reducing volume shrinkage rates and node displacement, indirectly enhancing magnetic levitation device stability. The maglev-injection molding collaborative system developed in this study achieves dynamic protection capabilities through non-contact operation and precise parameter regulation while extending equipment lifespan through node displacement control, providing an innovative technological pathway for fire protection equipment advancement.

### A summary of research progress on magnetic levitation, injection molding and related technologies

**Fig. 2:**
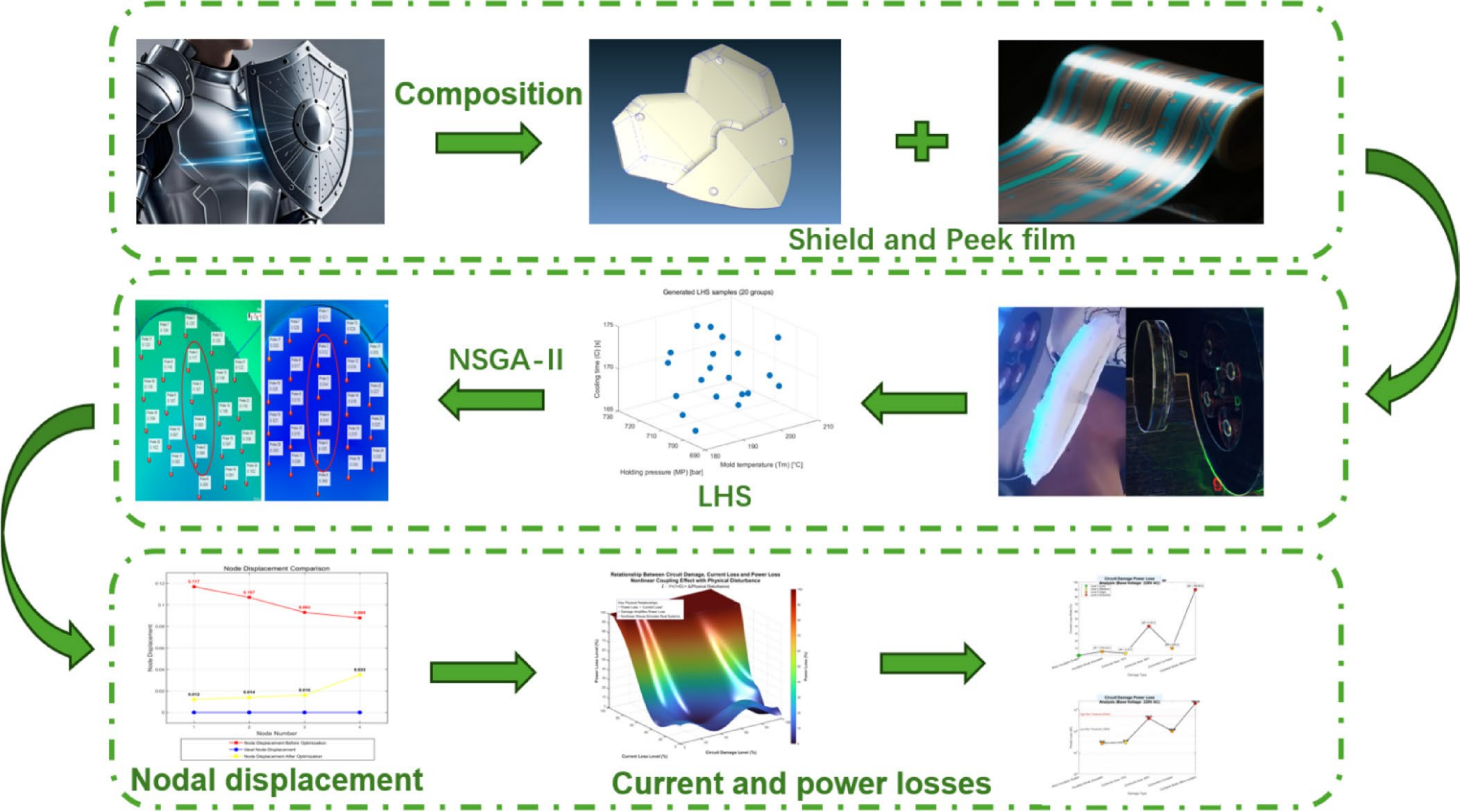
The optimization concept of the magnetic levitation shield study.

In the field of magnetic levitation technology, a series of research results have laid a crucial foundation for improving the performance of the device. The design of replacing permanent magnets with high-temperature superconducting (HTS) magnets enhances levitation stability by optimizing the magnetic field distribution, providing a new design idea for high-load maglev devices; By taking advantage of the rigidity of superconducting magnetic bearings and the superconducting pinning effect, stable levitation without active control was achieved, breaking through the stability bottleneck of repulsive magnetic levitation. The development of the coupling calculation model of lateral force and levitation force and the multimodal control architecture, through dynamic analysis and controller parameter optimization, significantly enhanced the system’s anti-interference ability and robustness. These studies have revealed the dynamic correlation mechanism of the force system of the magnetic levitation device, providing theoretical support for its stable application in firefighters’ shields. Innovations in injection molding focus on node displacement control and molding defect optimization. Virtual in-mold decoration (IMD) covering technology based on polyether ether ketone (PEEK) film enables rapid diagnosis and optimization of high-precision injection molding processes through mold exponential quantification and parameter simulation; Combined with Latin hypercube sampling and multi-strategy differential evolution algorithm, node displacement was reduced by 95.38 percent, ensuring circuit reliability; The damage detection-repair integrated solution builds a full-process closed-loop management from defect identification to functional recovery through film rupture characteristic localization and additive manufacturing repair. These advancements suggest that the combination of virtual simulation and intelligent algorithm-optimized parameters can effectively suppress forming defects such as node displacement and volume shrinkage. At the level of technology integration, in-mold electronics (IME) technology breaks through the single decorative function of traditional in-mold decoration (IMD), embeds sensors and circuits into film layers, and enables intelligent interaction through injection molding, advancing in-mold forming to dynamic perception. The NSGA-II and LHS algorithms are applied to injection molding parameter optimization. Through efficient sampling and multi-objective balancing, the volume shrinkage rate of the plastic part and the node displacement are simultaneously reduced, indirectly enhancing the stability of the magnetic levitation device. The maglev—injection molding collaborative system constructed in this study, through non-contact protection and injection molding parameter regulation, not only achieved the dynamic protection capability of the firefighter shield, but also extended the equipment life through node displacement control, providing an innovative path for the technological upgrade of fire protection equipment (As shown in Figure 2).

## Materials and methods

Polyetheretherketone (PEEK) is a special engineering plastic with excellent performance, which was successfully developed by ICI in the 1970s. With the rapid advancement of materials science, PEEK material has gradually become one of the core materials in high-end fields such as aerospace, mechanical manufacturing, and medical devices, thanks to its unique properties. With breakthroughs in high-precision molding processes, PEEK’s application scope has expanded further, gradually penetrating from traditional industrial fields to emerging industries such as electronics and energy. The surging global market demand for lightweight, heat-resistant and corrosion-resistant materials has further made PEEK one of the hotspots in materials science research.

The core characteristics of polyetheretherketone (PEEK) can be described as "lightweight, high strength, and resistance to extreme environments."

PEEK, with a density far lower than that of metals, offers mechanical strength close to that of metals, which makes it widely used in aerospace and protective fields as a preferred alternative to metals, not only significantly reducing the weight of equipment but also effectively reducing energy consumption^24^; PEEK material has tensile strength of 90-100 MPa, flexural strength of over 150MPa, stable performance under long-term dynamic load, and fatigue resistance superior to most engineering materials^25^; PEEK has a glass transition temperature of up to 143 °C, a melting point of 334 °C, can be used for a long time at 250 °C, and can withstand short-term temperatures up to 300 °C^26^. This provides a guarantee for firefighters to work in a fire environment.

PEEK possesses a glass transition temperature of 143°C and a melting point of 334°C, allowing for long-term use at 250°C and withstanding short-term thermal exposure up to 300°C. This provides the material basis for its application in extreme environments—specifically, the fire environment involving short-term exposure to temperatures above 300°C, which is the focus of this study concerning firefighters’ protective equipment. The exceptional stability of PEEK under this specific extreme thermal condition is crucial for ensuring the functional integrity of the protective equipment.

For quantitative performance evaluation of the protection system, this study establishes a parameterized definition of "fire environment." This environment refers to extreme thermal conditions with high-intensity radiant and convective heat, within a temperature range of 250°C to 400°C and heat flux density of 5-20 kW/m^2^. The analysis focuses on short-term exposure of 30 seconds to 5 minutes in this environment, emphasizing thermal degradation of polymer materials (e.g., PEEK) and consequent structural failure risks, rather than ablation under sustained flame.

PEEK, with a density far lower than that of metals, offers mechanical strength close to that of metals, which makes it widely used in aerospace and protective fields as a preferred alternative to metals, not only significantly reducing the weight of equipment but also effectively reducing energy consumption; PEEK material has tensile strength of 90-100 mpa, flexural strength of over 150MPa, stable performance under long-term dynamic load, and fatigue resistance of most engineering materials, providing a solid foundation for protective equipment manufacturing; PEEK has a glass transition temperature of up to 143°C, a melting point of 334°C, can be used for a long time at 250°C, and can withstand short-term temperatures up to 300°C. This provides a guarantee for firefighters to work in a fire environment.

With the continuous development of high-end manufacturing, the requirements for material properties are constantly increasing. PEEK material, with its lightness, high strength and excellent environmental adaptability, will occupy an irreplaceable position in protection and injection molding. With the continuous optimization of production technology and the reduction of costs, it is expected to become one of the key materials driving industrial upgrading.

**Fig. 3:**
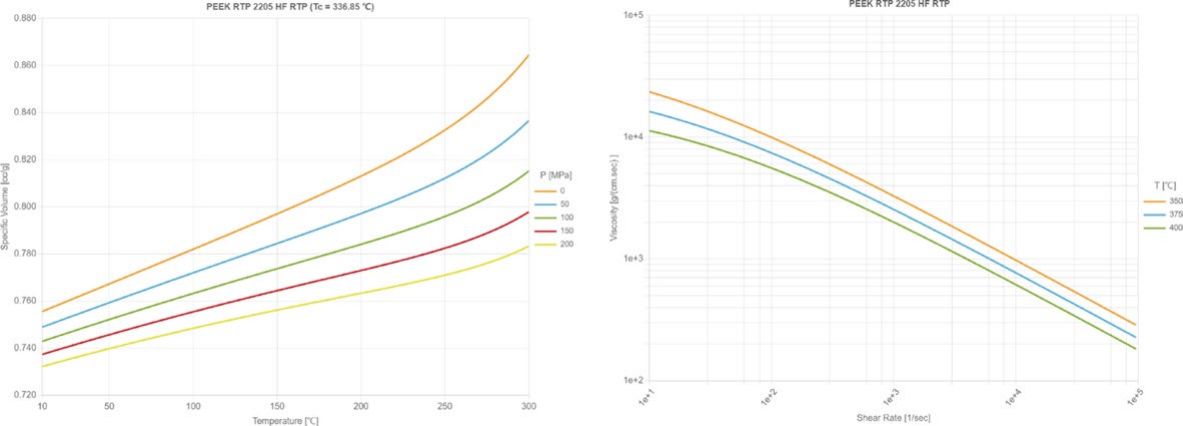
Diagram of PVT and viscosity of PEEK material.

Therefore, the formed shields and armors in this paper are made of PEEK. The low weight feature of the product reduces the workload of firefighters, while the high strength of protection ensures that firefighters’ safety is fully guaranteed during work. Figure 3 illustrates the key properties of PEEK material.

### Latin hypercubic sampling (LHS)

LHS is a widely used stratified sampling technique with obvious application value in various fields, which can maintain the representativeness of sampling points while effectively reducing computational costs. The core idea is to ensure uniform coverage of the sample in the multi-dimensional space through layering and random combination, while avoiding the clustering phenomenon that may occur in the traditional Monte Carlo method.

The basic principle is as follows: First, divide the range of values for each parameter into intervals of equal probability, ensuring that there is only one sampling point in each interval; Next, generate sample points in the multi-dimensional space by randomly arranging interval samples sampled in different dimensions. Compared with random sampling, LHS can cover the entire parameter space with a smaller sample size and avoid correlations among samples.

### LHS algorithm flow

Taking the extraction of N samples in d-dimensional parameter space 1 as an example:

1. Normalize the range of values for each dimension variable to [0,1] to improve the efficiency of subsequent data processing.
  1 $${\mathbf{x}}_{i}^{(norm)} = \frac{{{\mathbf{x}}_{i} - a_{i} }}{{b_{i} - a_{i} }},\quad \forall {\mathbf{x}}_{i} \in [a_{i} ,b_{i} ]$$
  Restore the generated samples to the actual range through inverse transformation:
  2 $$x_{i} = a_{i} + x_{i}^{{\left( {{\mathrm{norm}}} \right)}} \cdot \left( {b_{i} - a_{i} } \right)$$
2. For each dimension, divide the normalized interval [0,1] into N equal-width intervals:
  3 $$k = \left[ {\frac{k - 1}{N},\frac{k}{N}} \right),\quad k = 1,2, \ldots ,N$$
3. Randomly select a value as a sample based on a uniformly distributed random number m in each equal-width interval:
  4 $$x_{i}^{(k)} = \frac{k - 1}{N} + {\mathrm{m}} \cdot \Delta ,\quad {\mathrm{m}}\sim\;U(0,1)$$
  Randomly select a point in each interval, avoiding the aggregation of sampling points.
4. For each dimension, a random sequence πi is generated. The three samples are reorganized according to the random sequence to ensure the random and independent combination of subsequent multi-dimensional samples.
  5 $$\pi_{i} = {\mathrm{RandomPermutation}} (1,2, \ldots ,N)$$
5. Randomly arrange the samples from each dimension and combine them into sample points j in the d-dimensional space.
  6 $$j:\left( {x_{1}^{{\left( {r_{1} (j)} \right)}} ,x_{2}^{{\left( {r_{2} (j)} \right)}} , \ldots ,x_{d}^{{\left( {r_{d} (j)} \right)}} } \right),\quad j = 1,2, \ldots ,N$$

In injection molding, the quality of the product is closely related to the injection molding process parameters. Setting the parameters based on traditional experience is not only inefficient but also has a large error. By using Latin hypercube sampling, each process parameter is regarded as a dimension of the multi-dimensional space, and multiple sample points are selected to form multiple parameter combinations by taking advantage of its sampling characteristic of multiple coverage with fewer samples. Through mold flow analysis, the target product quality indicators, such as node displacement and volume shrinkage rate, are obtained. Based on the analysis of experimental data, combined with the response surface model and NSGA-II to optimize the injection molding parameter combination, the product quality is continuously improved.

### Non-dominated sorting genetic algorithm (NSGA-II)

NSGA-II is a genetic algorithm-based multi-objective optimization algorithm that, by simulating biological evolution processes, seeks a Pareto optimal solution set among multiple objectives (a solution set where no single solution is superior to other solutions on all objectives), and has significant application value in the field of parameter tuning.

The NSGA-II algorithm process:

1. Randomly generate the initial population At, of size M, through uniform distribution.
2. Calculate the value of each individual in the population At on the objective function, with the specific number of functions defined by the problem.
  7 $$f_{1} (x),f_{2} (x), \ldots ,f_{k} (x)$$
3. Randomly select two individuals x and y from At each time, compare the non-dominance level and the crowding degree (non-dominance level is given priority), select an individual as the parent, cross and mutate the parent, and produce Bt of size M among the offspring.
  Non-dominant rank: x dominates y (x<y) if and only if:
  8 $$\forall i \in [1,k],\;f_{i} (x) \le f_{i} (y)\quad \exists j \in [1,k],\;f_{j} (x) < f_{j} (y)$$
  Calculation of crowding (1.9) versus comparison (1.10) :
  9 $${\mathrm{CD}}\left( {x_{j} } \right) = \sum\limits_{m = 1}^{{\overline{k}}} {\frac{{f_{m} \left( {x_{j + 1} } \right) - f_{m} \left( {x_{j - 1} } \right)}}{{f_{m}^{\max } \left( {F_{i} } \right) - f_{m}^{\min } \left( {F_{i} } \right)}}}$$
  If x and y belong to the same front layer, select the individual with greater congestion:
  10 $${\mathrm{CD}}(x) > {\mathrm{CD}}(y) \Rightarrow x$$
  Crossover and variation to produce offspring:
  11 $$\beta = \left\{ {\begin{array}{*{20}l} {(2u)^{{1/(\eta_{c} + 1)}} ,} \hfill & {u \le 0.5} \hfill \\ {\left( {\frac{1}{2(1 - u)}} \right)^{{1/(\eta_{c} + 1)}} ,} \hfill & {u > 0.5} \hfill \\ \end{array} } \right.$$
  Parent p1, p2 produce offspring c1, c2 (1.12) :
  12 $$c_{1} = 0.5\left[ {(p_{1} + p_{2} ) - \beta |p_{2} - p_{1} |} \right],\quad c_{2} = 0.5\left[ {(p_{1} + p_{2} ) + \beta |p_{2} - p_{1} |} \right]$$
  (u~U[0,1], ηc is the cross-distribution index)
  Perturbing the offspring variable x (1.13) :
  13 $$\delta = \left\{ {\begin{array}{*{20}l} {(2u)^{{1/(\eta_{m} + 1)}} - 1,} \hfill & {u < 0.5} \hfill \\ {1 - \left( {2(1 - u)} \right)^{{1/(\eta_{m} + 1)}} ,} \hfill & {u \ge 0.5} \hfill \\ \end{array} } \right.$$
  (*ηm* is the variation distribution index, and Δmax is the variable range).
4. Combine the parent generation with the offspring generation Bt to form a new population Ct, with a size of 2 M.
5. Non-dominated sorting was performed on the Ct and it was divided into multiple fronts F1, F2… Fn, F1 as the non-dominated solution set, F2 as the suboptimal solution set, and so on.
6. Starting from F1, add the leading layer individuals to P (t + 1) in sequence until the total size exceeds M after adding Fi to a certain layer. For individuals in Fi, those with higher congestion levels are prioritized for supplementation.
7. Check if the maximum number of iterations has been reached; otherwise, return 2 and repeat.

Injection molding is a complex manufacturing process that involves the optimization of multiple parameters such as temperature, pressure, and time during the molding process to optimize multiple product quality objectives. NSGA-II is an efficient multi-objective optimization algorithm that can effectively solve injection molding parameter optimization problems and find the Pareto optimal solution set. In this paper, the objective function is to minimize the node displacement and volume reduction rate. Compared with the traditional single-objective optimization, NSGA-II has the advantage of multi-objective process parameter optimization. By combining the response surface model (RSM), NSGA-II is used to search for the optimal solution set of the mold temperature, holding pressure and cooling time.

## Case study

### Latin hypercube sampling (LHS)

In shield manufacturing, the setting of injection molding parameters has a significant impact on the quality characteristics of the product, and different combinations of parameters produce very different effects^27^. Therefore, in order to minimize the defects of the shield, it is necessary to optimize the injection molding parameters, by comparing and analyzing the molding results, and through optimization to obtain the best parameter combination.

**Fig. 4:**
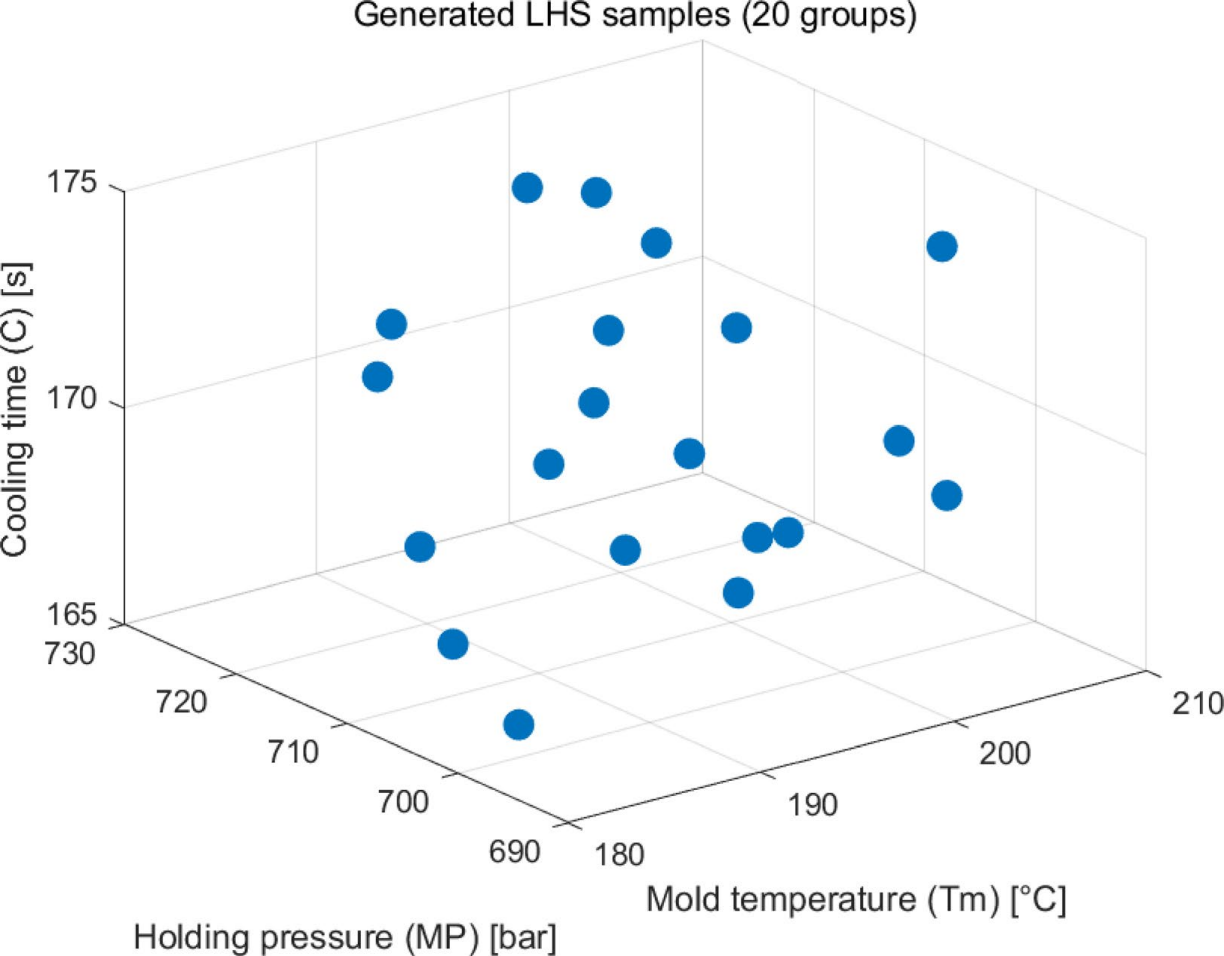
3D relationship diagram of 20 sets of process parameter combinations obtained by LHS.

Latin hypercube sampling has become the preferred sampling method in areas such as complex system modeling and uncertainty analysis, as it significantly improves spatial coverage uniformity and estimation efficiency while ensuring a low sample size through a layered strategy and dimensional independence. In this paper, wearable armor is used as the simulation model, and Moldex 3D is used for mold flow analysis. Mold temperature, holding pressure and cooling time are the study variables, and product node displacement and volume shrinkage rate are the objective functions. Twenty different injection molding parameter combinations were extracted and combined within the given parameter range by LHS (Table 1), as well as a 3D graph of the parameter combinations (Figure 4). At the same time, the simulation results of node displacement and volume shrinkage rate obtained from each parameter combination were recorded and analyzed and compared in order to gain a deep understanding of the relationship between the parameters and the results (Table 2).

**Table 1:** 20 combinations of LHS process parameters.

Group/parameters	Mold temperature (°C)	Holding pressure (MPa)	Cooling time (s)
001	203	697	174
002	194	711	172
003	191	690	170
004	198	720	173
005	188	722	170
006	205	725	171
007	200	729	172
008	182	705	167
009	183	700	165
010	189	695	171
011	181	707	174
012	192	713	169
013	206	719	165
014	201	693	169
015	197	716	166
016	208	709	168
017	185	698	173
018	209	726	168
019	187	715	167
020	196	702	166

**Table 2:** Simulation results of 20 groups of samples.

Group/result	Node displacement	Average	Standard deviation	Volume shrinkage rate %	Average	Standard deviation
001	0.013 ~ 0.816	0.338	0.127	7.386 ~ 1.680	− 3.347	1.391
002	0.006 ~ 0.526	0.231	0.098	7.538 ~ 1.859	− 3.586	1.376
003	0.001 ~ 0.491	0.221	0.095	7.237 ~ 1.526	− 3.295	1.388
004	0.001 ~ 0.590	0.258	0.102	7.685 ~ 2.006	− 3.697	1.376
005	0.002 ~ 0.498	0.214	0.094	7.667 ~ 2.005	− 3.762	1.365
006	0.008 ~ 0.963	0.372	0.156	7.783 ~ 2.105	− 3.735	1.385
007	0.013 ~ 0.653	0.286	0.111	7.815 ~ 2.151	− 3.815	1.371
008	0.006 ~ 0.481	0.209	0.092	7.381 ~ 1.742	− 3.543	1.367
009	0.005 ~ 0.481	0.209	0.092	7.330 ~ 1.656	− 3.468	1.377
010	0.004 ~ 0.491	0.217	0.094	7.303 ~ 1.602	− 3.375	1.378
011	0.014 ~ 0.362	0.192	0.067	7.062 ~ 0.697	− 4.083	1.719
012	0.003 ~ 0.500	0.223	0.096	7.555 ~ 1.895	− 3.620	1.364
013	0.020 ~ 1.003	0.395	0.171	7.696 ~ 2.026	− 3.645	1.382
014	0.006 ~ 0.484	0.224	0.093	7.514 ~ 1.818	− 3.626	1.444
015	0.047 ~ 0.477	0.226	0.084	7.525 ~ 1.768	− 3.510	1.440
016	0.004 ~ 0.475	0.213	0.090	7.464 ~ 1.795	− 3.659	− 1.430
017	0.019 ~ 0.524	0.245	0.087	7.322 ~ 1.697	− 3.448	− 1.369
018	0.001 ~ 0.431	0.213	0.060	7.159 ~ 1.687	− 3.667	− 1.334
019	0.028 ~ 0.779	0.389	0.174	7.482 ~ 1.765	− 3.730	− 1.458
020	0.041 ~ 0.821	0.411	0.167	7.511 ~ 1.787	− 3.742	− 1.436

**Fig. 5:**
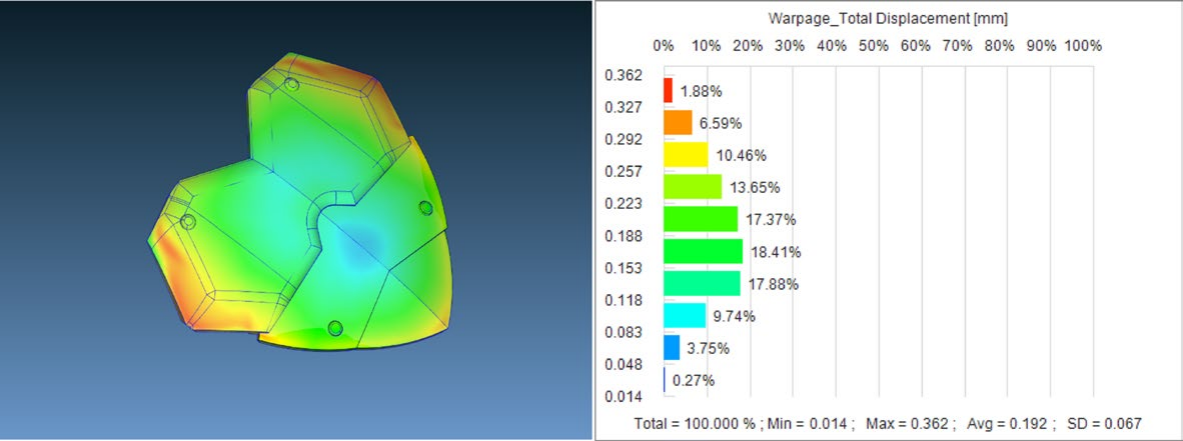
011 Group parameter combination node displacement model and bar chart.

**Fig. 6:**
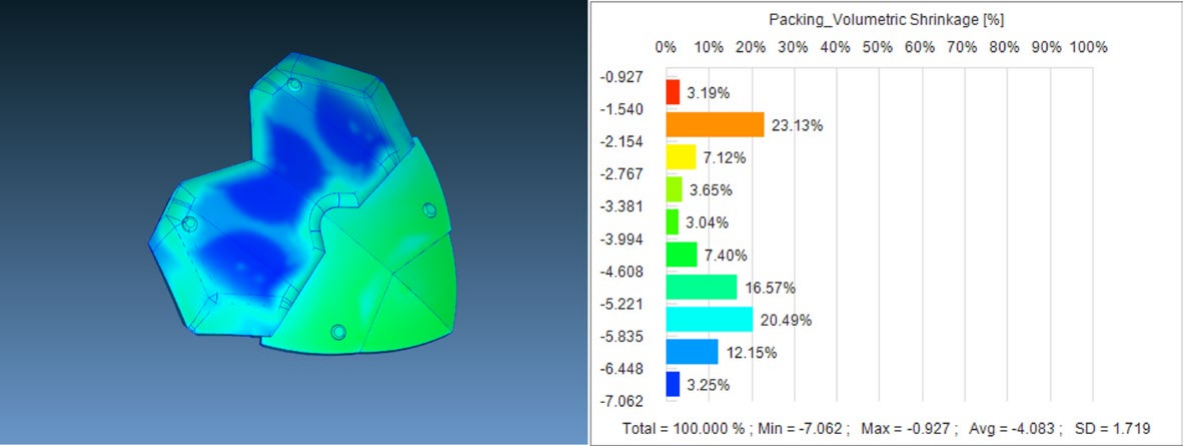
011 Model and bar chart of volume shrinkage rate for parameter combinations.

The initial results were analyzed with group 011 as an example, where the minimum node displacement was only 0.014mm, the maximum was 0.362mm, and the main node displacement distribution was concentrated between 0118 and 0.257mm, as shown in Figure 5. The minimum volume reduction rate was − 7.062%, the maximum was − 0.697%, and the host volume reduction rate distribution was concentrated between − 5.835 and − 4.608%, as shown in Figure 6. Node displacement and volume shrinkage rate have a significant impact on the stability of the IME circuit. Small node displacement and volume shrinkage rate can effectively reduce current loss, while better ensuring the stability of the circuit signal, providing good stability for the magnetic levitation device and better protection for firefighters at work. (The model diagram shows the situation of the target values at each position; The bar chart shows the distribution of target values.)

Simulation analysis has been conducted on 20 sets of sample data. In addition, the effect of pairwise interaction of the three parameters on node displacement needs to be studied to gain a deeper understanding of warpage and provide ideas for subsequent optimization directions.

**Fig. 7:**
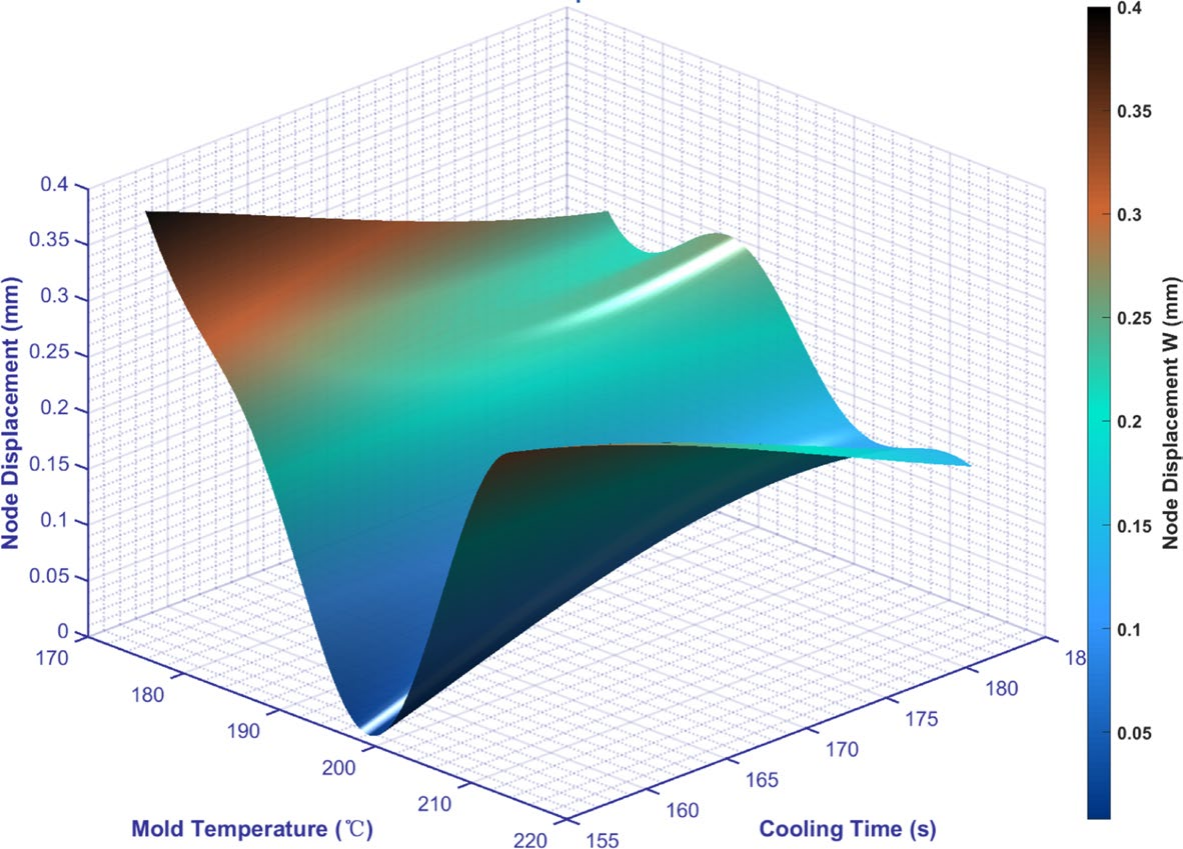
The effect of mold temperature and cooling time interaction on node displacement.

In injection molding, there is a significant interaction between mold temperature and cooling time on the amount of warpage (As shown in Figure 7), as shown below:

### Medium to low cooling time (155-170s)

Temperature from 170 to 200°C: The amount of warpage decreases gradually. Medium to low cooling times combined with appropriately elevated mold temperatures improve plastic fluidity and make shrinkage more uniform, thereby reducing warpage.

Temperature from 200 to 220°C: The amount of warping increases gradually. High temperatures may cause structural changes or degradation within the material, and these adverse effects cannot be fully offset even with short cooling times.

### Medium to high cooling time (170-180s)

Temperature from 170 to 200°C: The amount of warpage increases gradually. Longer cooling times may cause an increase in internal stress or other structural changes in the material, which in turn exacerbate warpage.

### Temperature from 200 to 220°C:

200°C to 215°C: Warpage is significantly reduced. The combination of high temperature and moderate cooling time makes the material shrink more evenly and reduces residual stress.

215°C to 220°C: Slight increase in warpage. It may be due to a slight degradation of the material caused by high temperatures or changes in certain properties that make it impossible to completely avoid the increase in warpage even with longer cooling times.

Overall, around 200°C is a critical temperature point, which is the lowest point of warpage at medium to low cooling times; At high cooling times, the amount of warpage decreased significantly in the range of 200°C to 215°C, but slightly increased again near 220°C. The choice of cooling time has different effects on different temperature ranges, and the cooling time needs to be adjusted according to the specific temperature range to optimize the warpage.

**Fig. 8:**
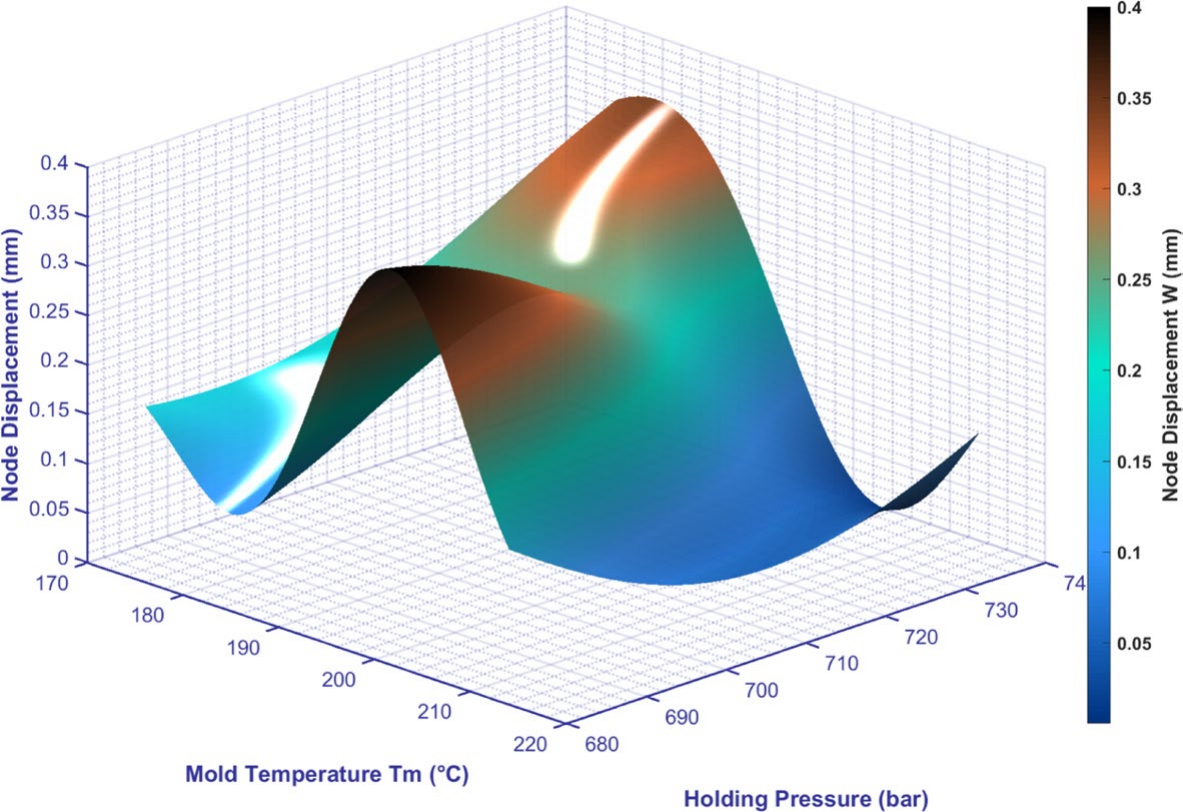
The influence of the interaction between mold temperature and holding pressure on node displacement.

There is a significant interaction between mold temperature and holding pressure on the warpage amount (As shown in Figure 8), as shown below:

### Low holding pressure (680-700bar)

Temperature from 170°C to 182°C: The amount of warpage gradually decreases, which indicates that in the low-temperature range, appropriately increasing the mold temperature helps to improve the amount of warpage. It may be because the increase in temperature improves the fluidity of the plastic, allowing the material to be distributed more evenly within the mold.

Temperature from 182°C to 200°C: the amount of warpage gradually increases to the peak. This may be due to the fact that as the temperature rises to a certain level, the material’s shrinkage properties and changes in internal stress begin to take the lead, resulting in an increase in warpage.

Temperature from 200°C to 210°C: the amount of warpage decreases gradually. This suggests that in the high temperature range, a moderate increase in temperature may allow the internal stress of the material to be released to some extent, thereby reducing the amount of warpage.

From 210°C to 220°C: the warpage increases gradually again. This could be due to excessive temperature causing material degradation or changes in internal structure, which exacerbated the warpage.

The variation in warpage under high holding pressure (700-740bar) is similar to that under low holding pressure.

It should be noted that at high temperatures (around 200°C), the warpage gradually decreases as the holding pressure increases from 680bar to 700bar. This suggests that at high temperatures, an appropriate holding pressure helps to reduce warpage. It may be because high holding pressure can better control the shrinkage and stress distribution of the material.

However, as the holding pressure continued to increase from 700bar to 740bar, the amount of warpage gradually increased. This could be due to an increase in internal stress in the material or other adverse effects caused by an excessively high holding pressure.

In injection molding processes, optimizing the combination of mold temperature and holding pressure is crucial for reducing warpage. It is necessary to find the best combination of process parameters based on specific production conditions to achieve high quality injection molding.

The observed critical transition in warpage behavior around a mold temperature of 200°C can be fundamentally attributed to the unique thermal properties of the PEEK material, specifically its glass transition temperature (Tg ≈ 143°C) and melting point (Tm ≈ 334°C). This temperature range (≈200°C) resides within the optimal window for PEEK’s crystallization kinetics.

When the mold temperature is significantly below 200°C, the polymer melt undergoes rapid cooling upon contact with the cooler mold surface. This rapid quenching near the surface can ‘freeze’ the polymer chains before they have sufficient time and mobility to relax and organize into a stable crystalline structure, leading to higher residual stresses and increased warpage.

As the mold temperature approaches and slightly exceeds 200°C, it provides a thermally favorable environment that is substantially above the Tg. This slower cooling rate allows for more uniform heat dissipation throughout the part thickness. Consequently, polymer chains gain enhanced mobility, facilitating stress relaxation and promoting the development of a more uniform and stable crystalline morphology. This reduction in internal stress and more homogeneous crystallization are the primary mechanisms behind the observed minimum in warpage at this critical temperature.

However, as the mold temperature continues to rise significantly above 200°C, approaching closer to the melting region, other competing effects may emerge. Excessive heat can potentially lead to subtle changes in the material’s viscoelastic properties or even initiate thermal degradation, which can reintroduce instability in shrinkage behavior and cause a slight rebound in warpage.

**Fig. 9:**
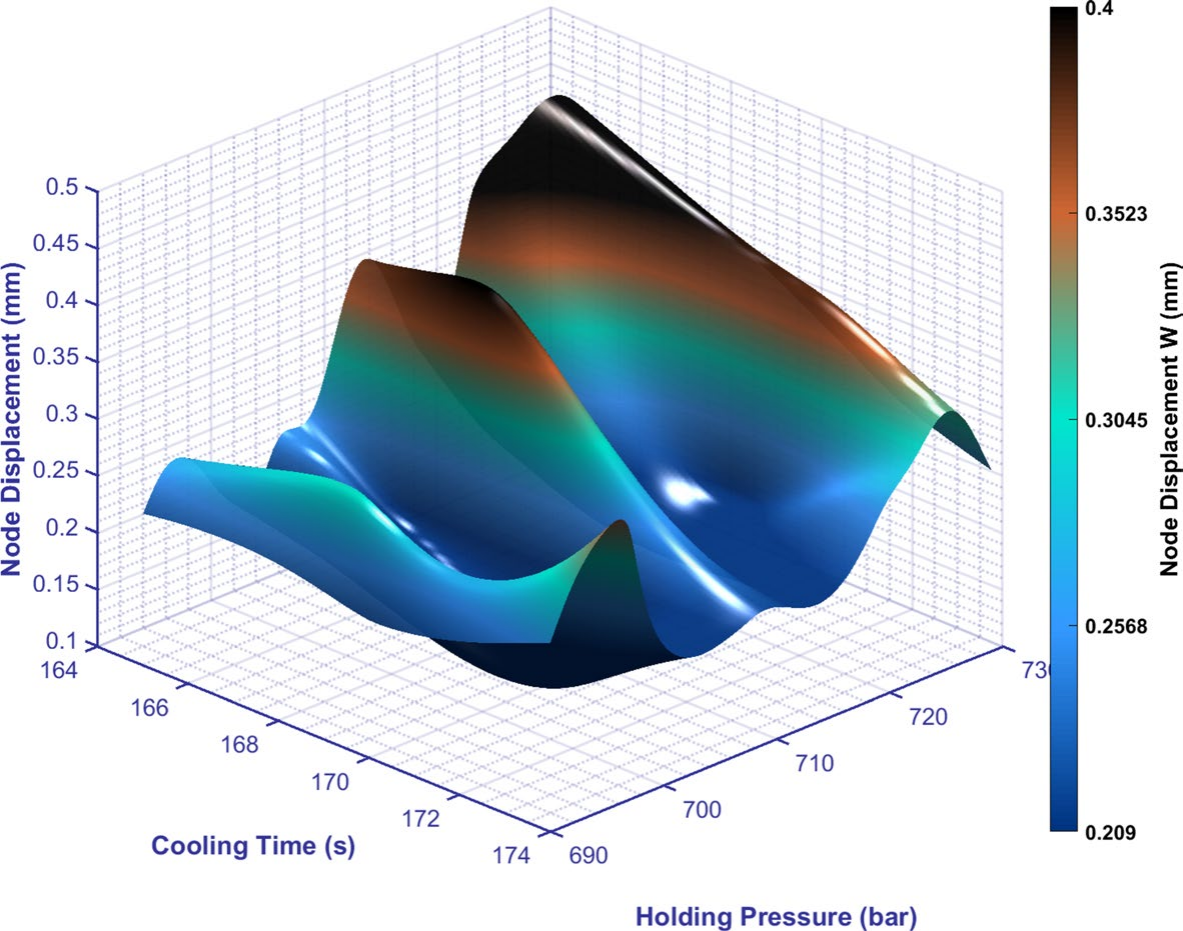
The effect of the interaction between cooling time and holding pressure on node displacement.

There is a significant interaction between cooling time and holding pressure on warpage (As shown in Figure 9), as shown below:

The entire graph shows a wavy pattern, indicating that the effects of cooling time and holding pressure on warpage are not linear but have multiple peaks and troughs. This complex interaction suggests that in injection molding, these two parameters need to be fine-tuned in order to achieve optimal warpage control.

1. Holding pressure at 690–695 bar:

The warpage increases slowly over the entire range of cooling time (164-174 seconds).

The low holding pressure cannot fully fill the mold, resulting in an uneven distribution of the material within the mold, an increase in residual stress, and thus a gradual increase in warpage.

2. When the holding pressure is 695-705 bar:

The warpage decreases slowly over the entire range of cooling time (164-174 seconds).

With a moderate increase in holding pressure, the material filling and compaction effect improved, the internal stress distribution became more uniform, and the warpage gradually decreased.

3. When the holding pressure is 705-710 bar:

When the cooling time is 164-168 seconds: The warpage increases rapidly.

The low cooling time causes the material to cool too quickly, and when the holding pressure reaches a certain value, it may cause excessive shrinkage of the material or internal stress concentration, thereby intensifying warpage.

When the cooling time is 168-174 seconds: the rate of increase in warpage gradually decreases.

As the cooling time increases, the material cools more evenly. Although the holding pressure is higher, the longer cooling time helps relieve some of the stress, which slows down the rate of increase in warpage.

4. When the holding pressure is 710-715 bar:

The warpage decreases slowly over the entire range of cooling time (164-174 seconds).

High holding pressure combined with moderately extended cooling time allows the material to be fully compacted and cooled evenly within the mold, further reducing the amount of warpage.

5. Holding pressure at 715-726 bar:

The warpage increases rapidly over the entire range of cooling time (164-174 seconds).

Excessive holding pressure may cause a significant increase in internal stress in the material, or even lead to material degradation or other adverse changes, resulting in a sharp increase in warpage.

6. When the holding pressure is at 726-730 bar:

During the entire cooling time range (164-174 seconds), the warpage gradually decreased.

Near the maximum holding pressure, there may be a certain pressure limit in the process system or a change in the rheological properties of the material, which instead causes the stress distribution of the material to rebalance and the warpage to decrease.

This complex interaction also reminds us that in actual production, merely adjusting a single parameter often fails to achieve the desired molding effect, and the synergistic effects of multiple process parameters must be considered comprehensively.

At this point, the study has formed 20 different parameter sample combinations by randomly sampling data within the specified parameter range through Latin hypercube sampling (LHS), and the corresponding target values, namely node displacement and volume reduction rate, have been simulated using Moldex 3D mold flow analysis software. At the same time, the effects of the three injection parameters on the node displacement were generated and the images were analyzed. In order to make the product have better attribute characteristics, it is necessary to further reduce the impact of defects through optimization algorithms such as NSGA-II.

### Non-dominated sorting genetic algorithm (NSGA-II)

NSGA-II is a classic multi-objective optimization algorithm. Its main content is to select the Pareto optimal solution that takes into account multiple objectives from the population through non-dominated sorting and crowding degree calculation. Compared with single-objective optimization algorithms, NSGA-II can solve multi-objective problems in injection molding more effectively. In this study, the product node displacement and volume shrinkage rate were used as objective functions, and the optimal solutions for both were obtained through NSGA-II, and the most suitable combination of process parameters was selected according to the actual needs. As a multi-objective optimization algorithm, weights need to be selected based on actual requirements. In this paper, the magnitude of node displacement is more important for the stability of circuit function. Based on the experimental results, higher weights will be given to node displacement to select the appropriate Pareto solution.

The MATLAB code implementing the NSGA-II algorithm for this optimization has been included in the Supplementary Material. The simulations were performed in MATLAB R2021a on a workstation equipped with an Intel Xeon E5-2680 processor and 64 GB of RAM.

**Fig. 10:**
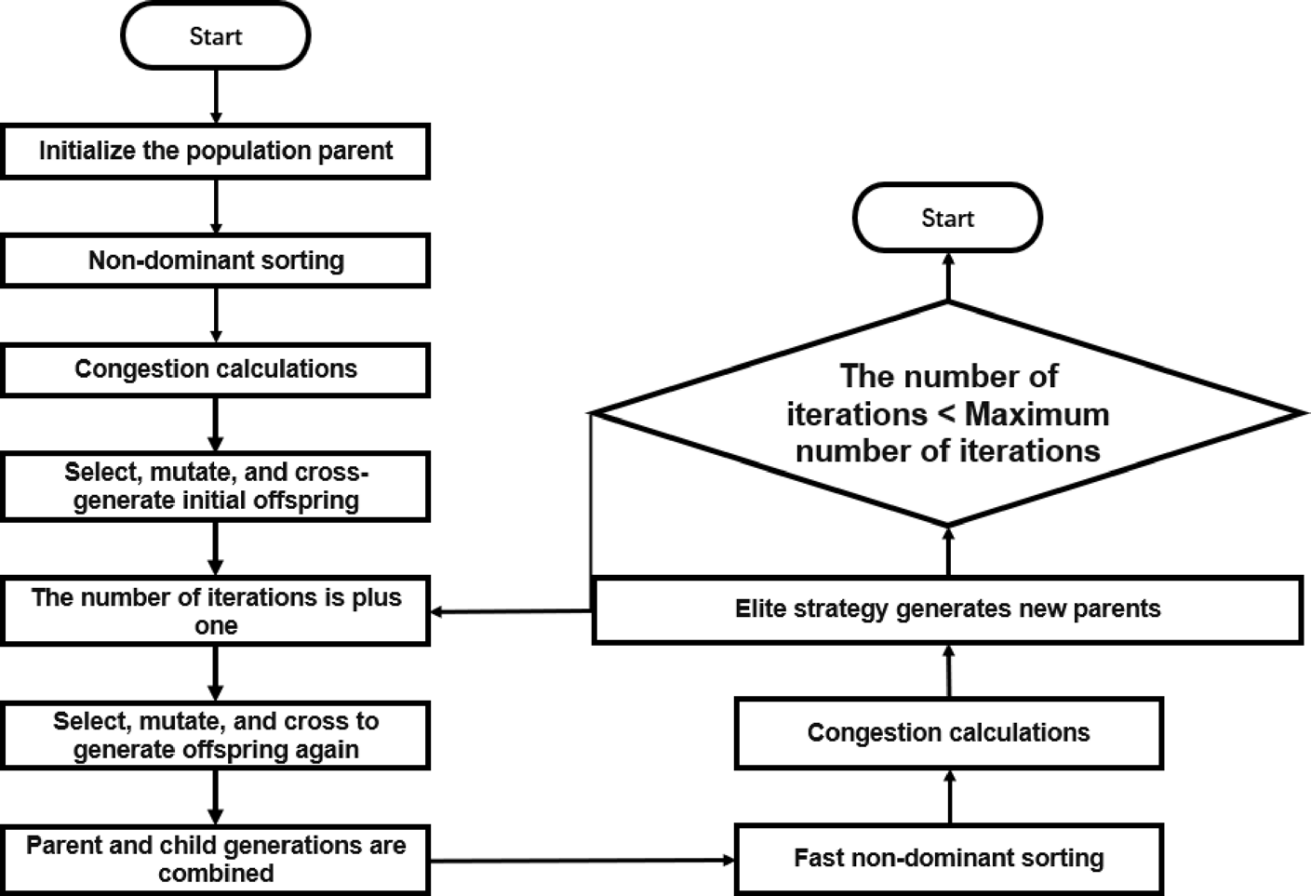
Flowchart of the optimization method.

**Fig. 11:**
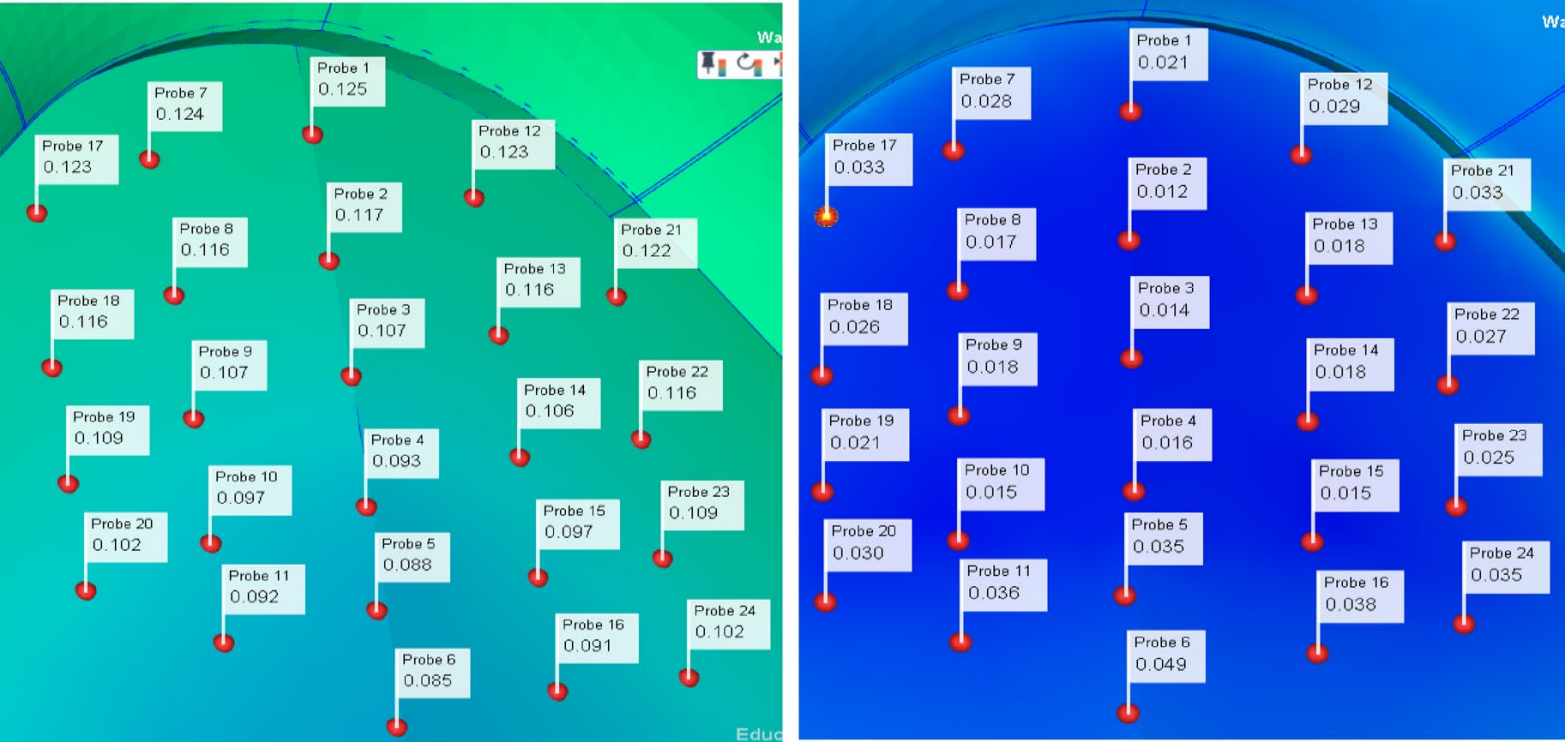
Graph of node displacement differences before and after optimization.

As shown in Figure 10, through the iteration and optimization of the original data by NSGA-II, the optimized parameter combination was obtained and inserted into the simulation software to obtain the optimized product. In order to observe more intuitively the changes in the displacement of product nodes before and after optimization, 24 nodes were selected as the research objects. The results before and after optimization were recorded and compared by NSGA-II method. And the optimization rate was calculated, as shown in Figure 11 and Table 3.

**Table 3:** Comparison of node displacement data before and after optimization.

Node numbering	Before optimization	After optimization	Optimization rate
1	0.125	0.021	83.2%
2	0.117	0.012	89.7%
3	0.107	0.014	86.9%
4	0.093	0.016	82.8%
5	0.088	0.035	60.2%
6	0.085	0.049	42.4%
7	0.124	0.028	77.4%
8	0.116	0.017	85.3%
9	0.107	0.018	83.2%
10	0.097	0.015	84.5%
11	0.092	0.036	60.9%
12	0.123	0.029	76.4%
13	0.116	0.018	84.5%
14	0.106	0.018	83.1%
15	0.097	0.015	84.5%
16	0.091	0.038	58.2%
17	0.123	0.033	73.2%
18	0.116	0.026	77.6%
19	0.109	0.021	80.7%
20	0.102	0.030	70.6%
21	0.122	0.033	80.0%
22	0.116	0.027	76.7%
23	0.109	0.025	77.1%
24	0.102	0.035	65.7%

According to the chart, it can be seen that the dislocations of all the selected nodes have improved significantly. Among them, node 2 has the maximum improvement rate of 89.7%, and node 24 has the minimum improvement rate of 65.7%. Based on the above data, not only the effectiveness of the combination of LHS and NSGA-II methods is verified, but also the quality of the product is effectively improved, providing a guarantee for the stable operation of the system.

## Results and discussion

The magnetic levitation shield is an innovative protective device composed of a magnetic levitation device and an IME circuit. The wearer can interact with the wearable armor based on the IME film to control the current size of the system and change the levitation ability of the magnetic levitation device, thereby enabling the shield to adjust the levitation distance according to different current sizes. It provides more flexible protection for firefighters.

Therefore, the stability of the current is very important for the implementation of the function, and in the injection molding process, the generation of various defects will affect the formation of the circuit, thereby indirectly reducing its stability, among which the node displacement of the product has a particularly obvious impact on the circuit. Ideally, the nodes do not shift and form in the original position, so the attachment of the IME circuit does not shift either; However, in actual production, the product may have defects due to various circumstances, causing the nodes to deviate from the intended position, resulting in a deviation in the formation of the circuit, affecting the current transmission, and ultimately affecting the implementation of the entire system function.

**Fig. 12:**
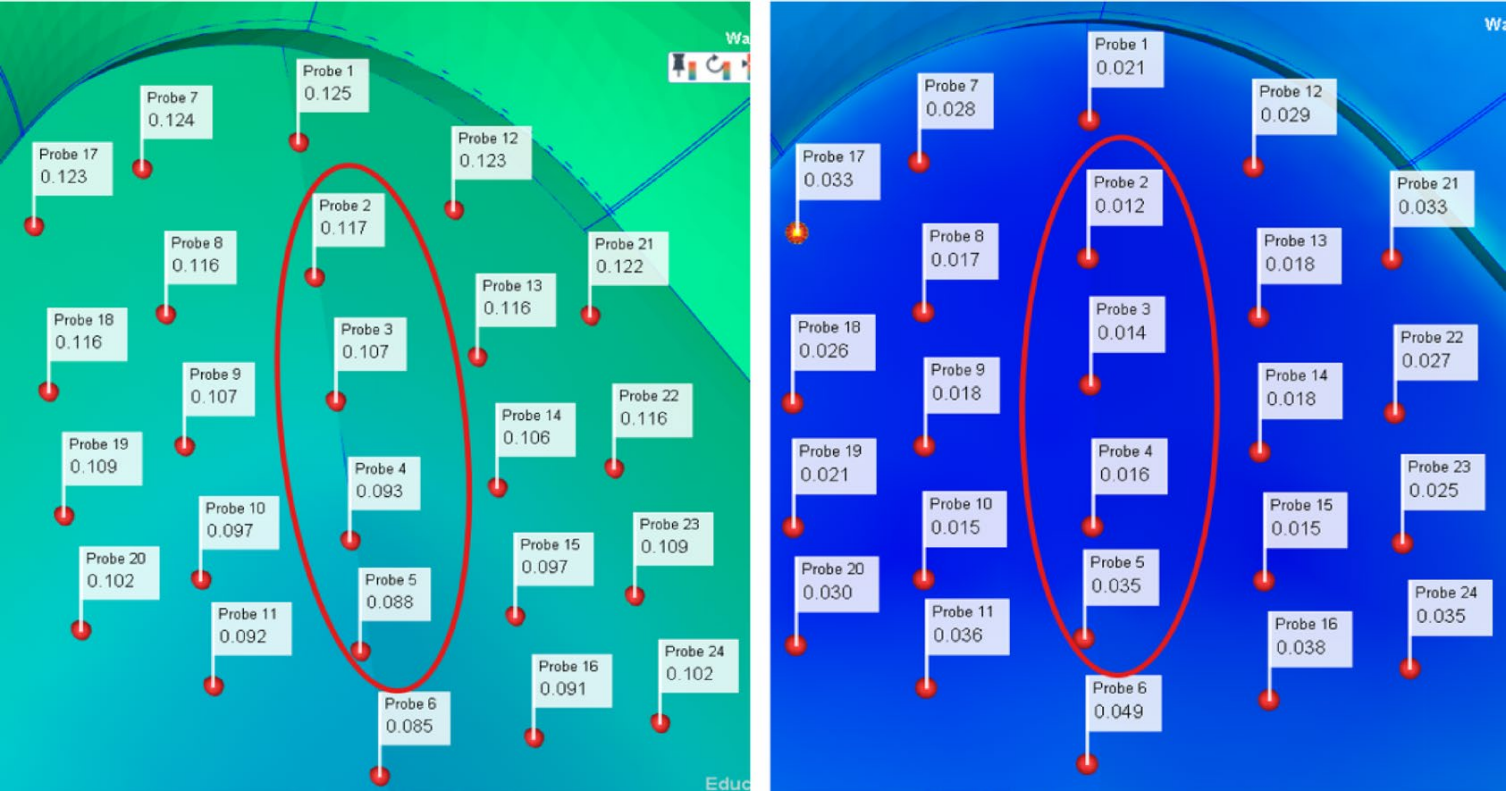
Four nodes are sampled as specific analysis research objects.

In order to discuss the changes before and after node displacement optimization in more detail, four nodes were selected from 20 nodes as specific research objects in this paper, as shown in Figure 12, to explore the influence of the above four nodes on the circuit before and after optimization.

**Fig. 13:**
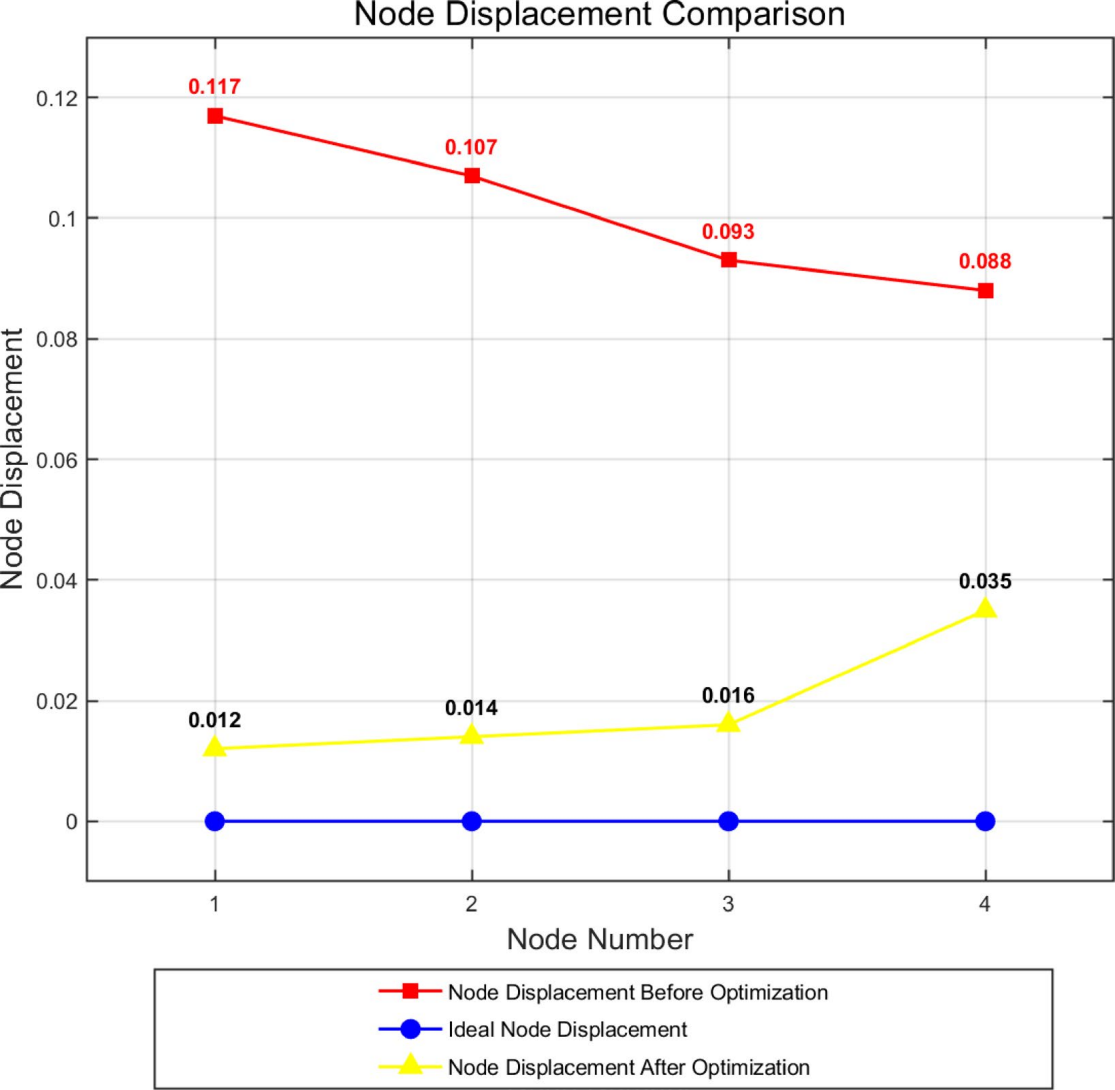
The ideal node displacement versus the difference in node displacement before and after optimization.

As shown in Figure 13, the horizontal axis represents the selection of four node labels, and the vertical axis represents the offset of each node. Blue represents an ideal thin-film IME circuit where the nodes do not offset, that is, the offset is 0; Red indicates relative displacement of the nodes before optimization during actual formation, and yellow indicates relative displacement of the nodes after optimization. It is easy to find that the displacement of the nodes after optimization is significantly less than that before optimization. The displacement of the nodes will cause corresponding changes in the current passing through, indirectly reducing the stability of the system. Through the optimization effect of the proposed research method, the displacement is effectively reduced. While verifying the effectiveness of the method, the stability and functionality of the system have been improved.

**Fig. 14:**
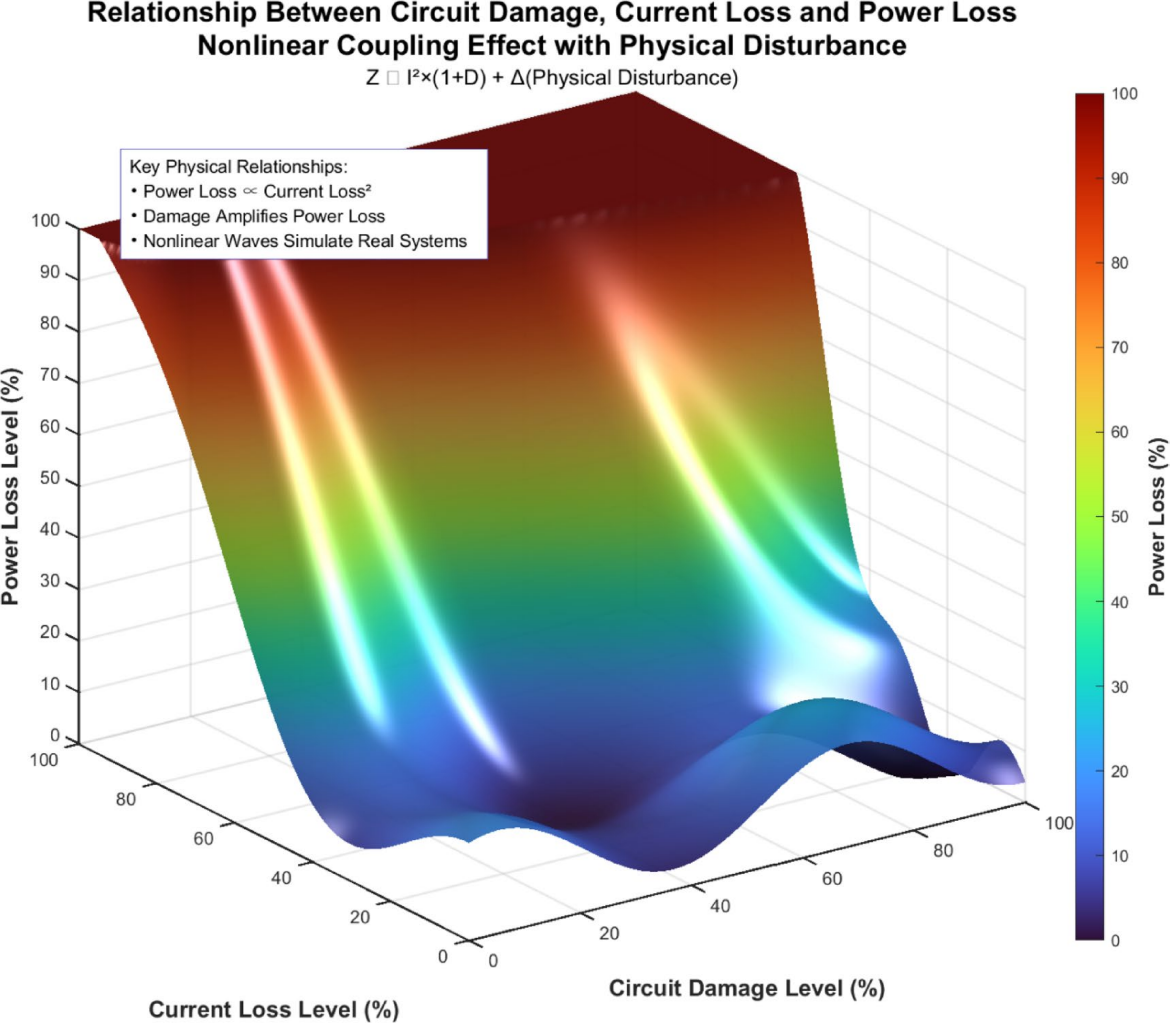
Three-dimensional graph of circuit damage with current and power damage.

Studying the impact of circuit damage caused by node displacement on current loss rate and power loss, as well as safety risk level, is of great significance for the safety of workers in the actual working environment. This paper discusses circuit damage and current loss rate and power loss from two dimensions, First, the nonlinear relationship among circuit damage degree (X-axis), current loss degree (Y-axis), and power loss degree (Z-axis) is visually presented through three-dimensional surface diagrams, as shown in Figure 14.

**Fig. 15:**
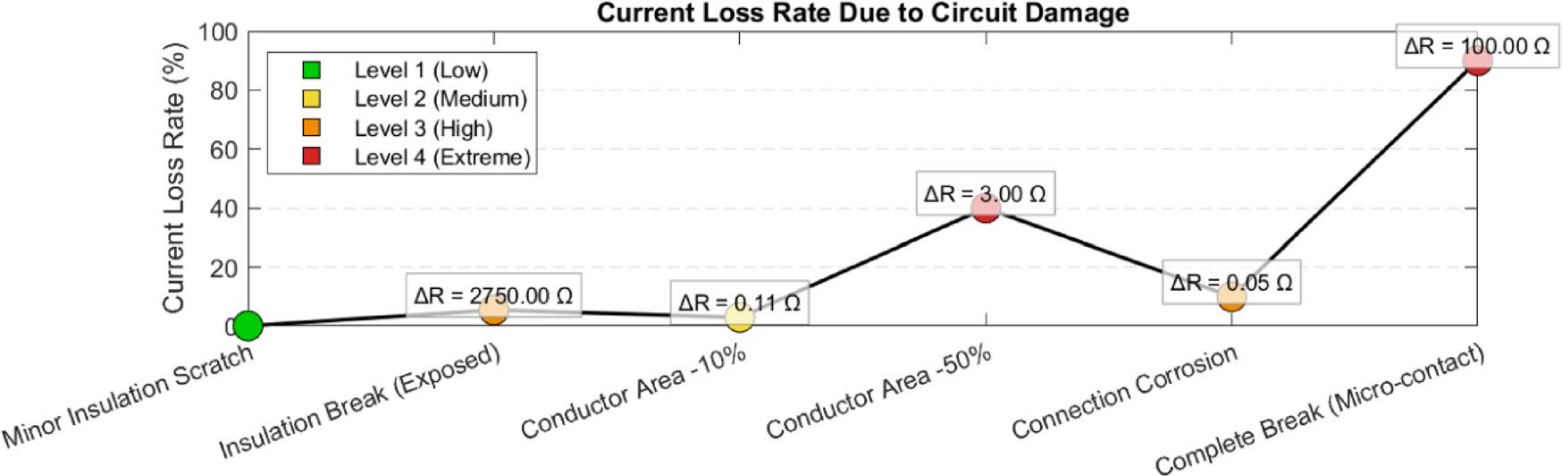
Graph of circuit damage versus current loss rate.

**Fig. 16:**
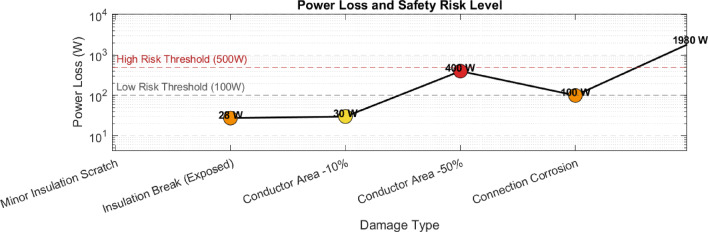
Diagram of circuit damage versus power loss.

To gain a more specific understanding of the relationship between circuit damage and current and power damage, the study conducted a detailed analysis by converting the three-dimensional graph into a two-dimensional graph, as shown in Figures 15 and 16, to analyze the influence mechanism and risk level of different types of circuit damage.

The current loss rate reflects the extent to which the current transmission efficiency of a circuit decreases due to damage, and its core mechanism is directly related to Ohm’s law (*I*=*RV*). When damage occurs in a circuit, the equivalent resistance *Rdamage* increases, resulting in additional current loss at the point of damage. Here is a comparison of measured data for six typical damage types (Table 4).

**Table 4:** Analysis of the correlation between damage type and current loss.

Damage type	Resistance increment (ΔR, Ω)	Current loss rate (%)	Risk level	Color identification
Slight insulation scratches	0	0.1	1 (Low)	Green
Insulation scratch (exposure)	2750	5.5	3 (High)	Orange
Conductor cross-sectional area − 10%	0.11	3.0	2 (Middle)	Yellow
Conductor cross-sectional area − 50%	3.0	40.0	4 (Extremely high)	Red
Corrosion of connection points	0.05	10.0	3 (High)	Orange
Complete fracture (micro-contact)	100	90.0	4 (Extremely high)	Red

### Low-risk damage (Level 1-2)

Slight insulation scratch, due to the insulation layer not being completely damaged, the leakage current is minimal and the current loss is negligible;

Conductor cross-sectional area − 10% and connection point corrosion, although the resistance increment is small (0.05-0.11Ω), will gradually escalate to high-risk damage due to the accumulation of Joule heat over long-term operation (for example, for every 0.01Ω increase in contact resistance during connection point corrosion, the temperature may rise by 5-8°C).

### High risk of damage (Level 3-4)

Insulation damage (exposure) causes the conductor to come into direct contact with air or foreign objects, creating a "hidden short circuit" path, resistance suddenly increases to 2750Ω, current loss rate up to 5.5%, potentially causing intermittent leakage faults;

When the cross-sectional area of the conductor is − 50%, the resistance is inversely proportional to the cross-sectional area (*R*∝*S1*), the resistance increases fourfold, the current is forced to split, and the loss rate surges to 40%, at which point the circuit is close to a "half-open" state;

When completely broken (micro-contact), a high-resistance arc is formed between the breakpoints, with a current loss rate of up to 90%, and the remaining weak current is conducted through air ionization or the oxide layer, which could cause arc discharge at any time.

Power loss (*P*=*I2*⋅*Rdamage*) is squared with resistance, causing it to increase logarithmically with the severity of damage. In the second subgraph, the vertical axis uses a logarithmic scale (1W-2500W), which clearly shows the power differences for different types of damage:

Minor insulation scratch: Power loss 0W, no substantial impact on the system;

Insulation damage (exposure) : Power loss 27.5W, corresponding to the low-risk threshold (100W);

Conductor cross-sectional area—50% : Power loss 400W, close to the high risk threshold (500W), at which point the temperature at the damage point can reach above 150°C (assuming ambient temperature 25°C, heat dissipation coefficient 10W/(m^2^ K));

Complete breakage (micro-contact) : The power loss is 1980W, which is 3.96 times the high-risk threshold. The instantaneous temperature at the breakage point can reach the melting point of copper (1085℃), which is sufficient to ignite the surrounding plastic or grease.

The two dotted lines in the figure (100W low risk threshold, 500W high risk threshold) `mark the “red line” for the safe operation of the circuit:

Low risk range (<100W) : If the connection point corrosion (100W) does not pose a direct threat to safety for the time being, the contact point temperature should be detected by infrared thermal imaging (normally < 60°C);

High-risk zone (>500W) : If the cross-sectional area of the conductor is − 50% (400W) and it is completely broken (1980W), the hazard must be eliminated through power-off maintenance to avoid electrical fires caused by overheating.

### Control circuit damage and power loss through the node displacement suspension shield

Magnetic levitation shields, as precision protective equipment, have performance stability subject to the synergistic effects of multiple dimensions. Product defects are mainly determined by human operation, environmental conditions and process parameters, among which process parameters can be regulated in a targeted manner through systematic optimization. Based on this feature, this paper proposes a multi-objective optimization strategy that integrates Latin Hypercube sampling (LHS) with the non-dominated sorting genetic algorithm (NSGA-II). By constructing a parameter optimization model to dynamically adjust the key process parameters of injection molding, it achieves precise suppression of product node displacement defects while covering a wide range of design Spaces. Reduce the potential risk of the circuit system at the structural level. The reliability of the circuit system is strongly associated with the node displacement of the shield structure. When the shield structure undergoes node displacement, the embedded circuit undergoes physical damage due to deformation, which leads to a nonlinear increase in current loss rate and power loss.

**Fig. 17:**
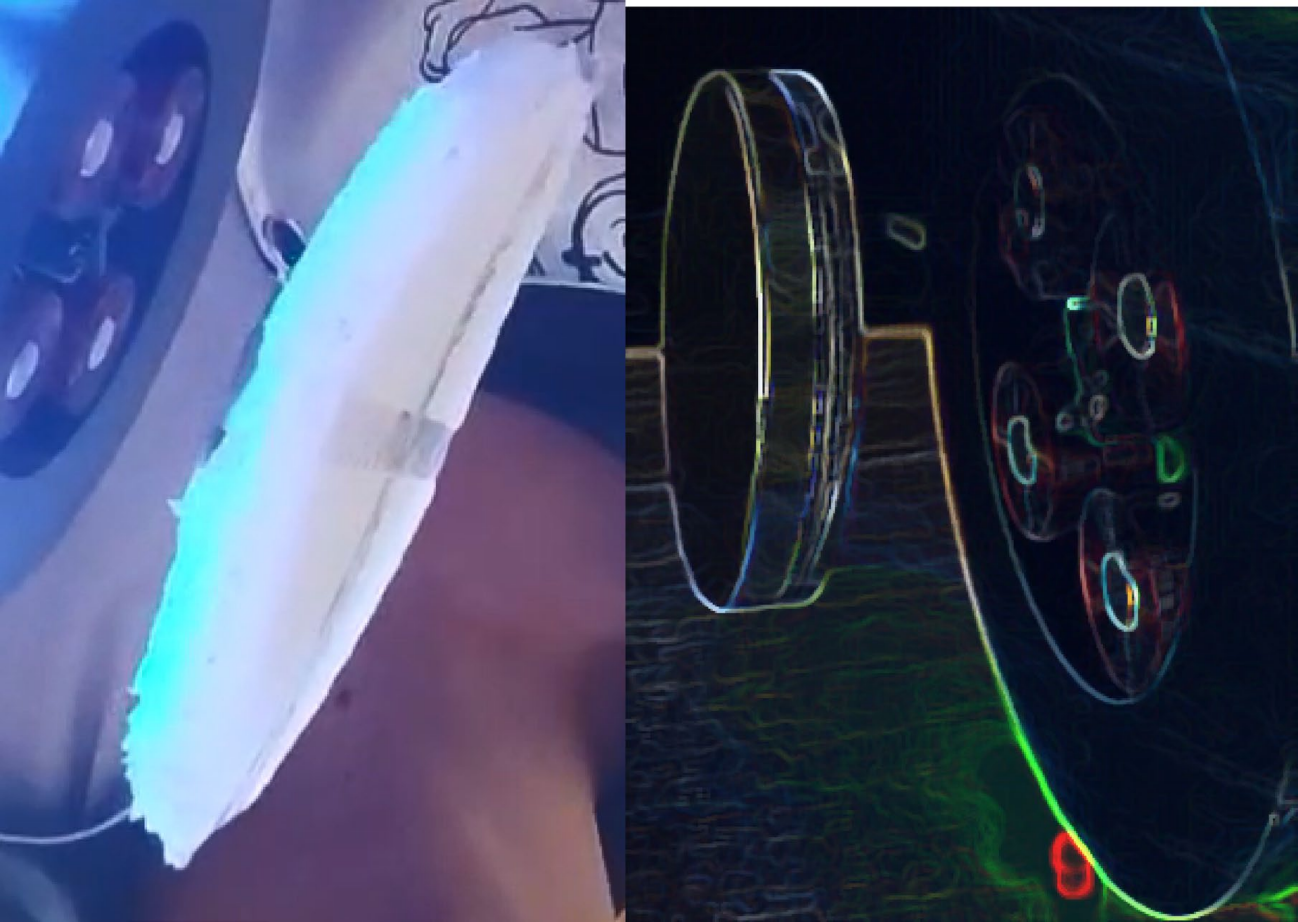
A suspended shield that controls circuit damage and power loss through node displacement.

The study, as shown in Figure 17, indicates that the impact of circuit damage on electrical performance indicators is significantly hierarchical: different degrees of damage result in differentiated responses of electrical losses, and this effect is not linearly superimposed but presents complex nonlinear changes as the degree of damage increases. This characteristic requires the introduction of a dynamic monitoring mechanism in shield design—by integrating micro-sensing technology to achieve real-time collection of key parameters, providing data support for the early identification of circuit hazards, and thereby optimizing the accuracy of maintenance strategies. In engineering practice, the optimization of the suspended shield should follow the principle of "prevention first, hierarchical handling" and build a multi-dimensional safety protection system. In terms of the design dimension, through material mechanics simulation and structural optimization, the anti-deformation ability can be enhanced while ensuring the protective performance; In terms of operation and maintenance, a risk level assessment system is established based on the correlation analysis of damage type and electrical loss, and targeted protection is implemented for high-risk areas; The monitoring dimension achieves precise control of process parameters through the combination of advanced simulation technology and real-time sensing technology, effectively avoiding circuit damage caused by node displacement, thereby reducing the probability of safety accidents and economic losses.

### Performance comparative analysis with traditional and standalone algorithms

To highlight the advantages of the LHS-NSGA-II integrated paradigm adopted in this study, it is necessary to compare it with traditional methods. As directly running a standalone NSGA-II algorithm on the high-fidelity model is computationally infeasible, we conduct the following qualitative comparison based on literature and computational principles:

### Comparison with traditional trial-and-error methods

Traditional methods heavily rely on engineer experience and typically optimize only one objective at a time or transform multi-objectives into a single objective through subjective weighting. This approach is not only inefficient but also highly prone to getting stuck in local optima, unable to systematically explore the design space. The method in this study achieves a paradigm shift from "experience-driven" to "data-driven," capable of automatically and globally providing a set of Pareto optimal solutions.

### Comparison with standalone NSGA-II algorithm

This is the most critical comparison. For a typical high-fidelity injection molding simulation (assuming one analysis takes 1 hour), a standalone NSGA-II algorithm typically requires 10^3 to 10^4 evaluations to converge, implying a total computation time exceeding one year, which is computationally infeasible in engineering practice. In contrast, this integrated paradigm uses LHS for a small number of initial samples (e.g., 10^2) to build a surrogate model, and then runs NSGA-II on the model that evaluates in milliseconds. This reduces the total computation time from the order of 10^4 hours sharply to the order of 10^2 hours, achieving an order-of-magnitude improvement in efficiency. This is the primary justification for the existence of this method.

Although this efficiency gain comes at the cost of introducing surrogate model uncertainty, this study effectively controlled this risk through high-quality LHS sampling and response surface model construction, as evidenced by the significant improvement in the optimization results.

This study addresses the fundamental challenge of optimizing *circuit node displacement*—a critical yet highly localized phenomenon in IME systems. For such specific, micro-scale geometric issues, the integrated methodology of *LHS-based sampling and NSGA-II optimization* represents the most efficient and targeted approach. The computational framework leverages high-fidelity simulation to generate a comprehensive dataset, enabling the algorithm to explore the parameter space in a way that is both systematic and cost-prohibitive to achieve through physical experimentation alone. While physical prototyping serves as an ultimate validation step in engineering, the precision of modern injection molding simulation in predicting displacement fields is well-established. Therefore, the methodology presented herein provides a robust and sufficient solution for the stated objective. Future work could, nevertheless, involve fabricating key geometric features to corroborate the numerical findings at minimal additional cost.

## Conclusion

This study developed an integrated optimization methodology combining Latin Hypercube Sampling (LHS) and the Non-dominated Sorting Genetic Algorithm II (NSGA-II) to address the injection molding quality challenges in firefighters’ magnetic levitation shields. The principal findings are summarized as follows:

1. Validation of methodological effectiveness: The LHS-NSGA-II fusion strategy successfully reduced node displacement by 65.7% to 89.7%, significantly suppressing product physical deformation. This method provides an effective approach for handling complex optimization problems involving multiple coupled process parameters.
2. Resolution of the core issue: A strong correlation between node displacement and the performance of IME circuits was established. Post-optimization, the risk of circuit damage was markedly reduced, and current transmission efficiency increased by over 83%, fundamentally ensuring the stable operation of the core magnetic levitation function.
3. Demonstration of a promising technical solution: This research not only provides a parameter optimization workflow superior to traditional empirical methods but also ultimately delivers a magnetic levitation shield prototype that combines non-contact dynamic protection with high intrinsic reliability, offering a new technical pathway and practical reference for developing advanced firefighting equipment.

## Supplementary Information

Below is the link to the electronic supplementary material.

Supplementary Material 1

## Data Availability

The authors declare that the data supporting the results of this study are available in the paper. If any raw data files in other formats are required, they can be obtained from the corresponding author upon reasonable request.
